# [Retracted] Cardioprotective effect of notoginsenoside R1 in a rabbit lung remote ischemic postconditioning model via activation of the TGF‑β1/TAK1 signaling pathway

**DOI:** 10.3892/etm.2025.12793

**Published:** 2025-01-03

**Authors:** Zhi-Ru Ge, Mao-Chun Xu, Yu Huang, Chen-Jun Zhang, Je Lin, Chang-Wu Ruan

Exp Ther Med 11:2341–2348, 2016; DOI: 10.3892/etm.2016.3222

Following the publication of this paper, it was drawn to the Editor’s attention by a concerned reader that, for the experiments showing hematoxylin and eosin staining of lung tissue tissues in Fig. 2 on p. 2344, the following duplications of data were apparent: i) panels A (control group, left lung) and B (control group, right lung) appeared to show exactly the same data; ii) these same data were centrally located in panel C (left lung, RIP group), at a different magnification; and iii) panels D (right lung, RIP group) and E (left lung, NG-R1 group) appeared to show the same data at different magnifications.

The Editorial Office have conducted an independent assessment of these data, and can verify that the concerns raised by the reader were valid. Therefore, in view of the large number of duplications of data that have been identified within Fig. 2, the Editor of *Experimental and Therapeutic Medicine* has decided that this paper should be retracted from the Journal on account of a lack of confidence in the presented data. The authors were asked for an explanation to account for these concerns, but the Editorial Office did not receive a reply.The Editor apologizes to the readership for any inconvenience caused.

